# Assessing equity in benefit distribution of government health subsidy in 2012 across East China: benefit incidence analysis

**DOI:** 10.1186/s12939-016-0306-z

**Published:** 2016-01-20

**Authors:** Mingsheng Chen, Andrew J. Palmer, Lei Si

**Affiliations:** School of Health Policy & Management, Nanjing Medical University, Hanzhong Road 140, Nanjing, 210029 Jiangsu Province PR China; Menzies Institute for Medical Research, University of Tasmania, Medical Science 1 Building, 17 Liverpool St (Private Bag 23), Hobart, TAS 7000 Australia

**Keywords:** Government health subsidy, Benefit distribution, Equity, Concentration index

## Abstract

**Background:**

Improving the equitable benefit distribution of government health subsidies, particularly among the country’s poorer socioeconomic groups, is a major goal of China’s healthcare sector reform.

**Methods:**

Benefit incidence analysis was employed to measure the distribution of government health subsidies by income quintile. The concentration index (CI) of different levels of health care facility in urban and rural areas was calculated. A household survey complete through multistage stratified sampling was conducted in 2013 in urban areas (16,908 respondents) and rural areas (19,525 respondents).

**Results:**

The overall CI for urban patients was 0.1068 for outpatient care and 0.1237 for inpatient care. For outpatient care, the CI was 0.0795, 0.0465 and 0.3456, respectively, at primary, secondary and tertiary health care facilities; for inpatient care, the CI was −0.2179, 0.0752 and 0.2883 at the corresponding facility levels. The overall CI for rural outpatients was −0.0659 and 0.0036 for inpatients. For outpatient care, the CI was −0.0818, 0.0567 and 0.0271 at primary, secondary and tertiary facilities, respectively; for inpatient care, the CI was −0.0050, 0.0084 and 0.0252 at the corresponding facility levels.

**Conclusions:**

China’s primary level health care facilities were found to have a more equitable benefit distribution of government health subsidies than the secondary- and tertiary- level facilities. Increased government budget allocations and insurance imbursement rates, and the provision of technical support and qualified medical staff to lower-level hospitals were key factors. However, the provision of equal subsidies to all socioeconomic levels was found to be a potential threat to the equity of government health subsidy distribution.

## Background

Equity in benefit distribution of government health subsidies has recently become a hot topic in Asia. The existing literature has demonstrated that better-off individuals receive more public health subsidies than the poor in many Asian countries and regions that rely heavily on out-of-pocket (OOP) payment. Researchers have also suggested that the poor receive fewer subsidies because they simply cannot afford to pay and so forego treatment [1–3].

Correspondingly, in China, market-oriented economic reform has been accompanied by reductions in state participation in the health sector, and government subsidies of health care facilities have been decreasing since the early 1980s. Along with the decentralization of financial responsibility for managing health care facilities, government expenditure on health has declined rapidly [4, 5]. For example, government funding accounted for more than 30 % of revenues in public hospitals in the early 1980s, but it shrank to 6.29 % by 2006 [6]. Although government funding was reduced, China’s health care facilities were free to provide any type of medical care and to charge for most medical goods in order to address deficits in operational cost and to provide incentives to the facilities and their medical staff [7]. However, direct payment and profit-seeking behavior promoted unnecessary treatment and aggressive drug sales in hospitals, resulting in increased OOP expenditures for the patients [8, 9].

Changes in the government health budget have also influenced access to health care and the distribution of government health subsidies among different socioeconomic groups [10]. The coverage and compensation offered by health insurance add to the inequity of government subsidies because the rich (those in the upper socioeconomic class) living in both urban and rural areas are preferentially covered. Low coverage within poorer socioeconomic groups and high reimbursement rates for the rich have led to inequitable differences in the utilization of health care and in access to subsidies between the rich and the poor [11, 12].

Confronted with these inequalities, efforts have recently been made to improve the utilization of healthcare subsidies across different socioeconomic groups. Since the start of China’s health care reform in 2009, government subsidies of health care facilities have increased, and have succeeded in maintaining relatively acceptable prices for medical services [13]. Affordable health care prices maintained by local price bureaus have made health services financially accessible to patients. The difference between low health care prices and high costs is compensated by government health subsidies, and patients benefit from the subsidy whenever they consume medical services in health care facilities [14]. Low-income and vulnerable groups should be of the highest priority for government health subsidies; however, little is known about the impact of the recent reforms on the previously inequitable distribution of health care benefits. Thus, a proper assessment of the benefit distribution of government health subsidy among different income groups is needed in order to help policy makers develop financial risk protection strategies to ensure that the poor benefit from government health subsidies.

The structure of China’s health care system is divided into three levels, both in urban and rural areas. Medical care is delivered by community health centers (CHCs), district hospitals (DHs) and municipal hospitals (MHs) in urban areas, which are primary, secondary and tertiary levels of health care facilities, respectively. It is correspondingly delivered by village clinics (VCs), township health centers (THCs) and county hospitals (CHs) in rural areas. In order to explore the differences in distribution equity, the benefit distribution of government health subsidies is analyzed by the level of health care facilities in this paper.

The remainder of the paper is organized in the following way: the Method section describes the study’s data sources and the procedure of benefit incidence analysis (BIA); data about socioeconomic and health status from the national health investigation are then critically analyzed and evaluated in the Results section. The final section discusses these results and attempts to draw some conclusions concerning the broader, international lessons that might be inferred from the Chinese experience.

## Methods

### Data sources

The study data were collected through a household survey conducted in Jiangsu province, East China, in 2013, and thus were representative of conditions in 2012. Jiangsu, located in the east of China, is a developed province with a population of more than 79.60 million people—51.91 million in urban areas and 27.69 million in rural areas [15]. The survey was administered in 18 cities and counties, and participants were selected using a multistage stratified random sampling method. In each city or county, 10 communities or villages were selected by economic level and geographic distribution. In each community and village, 70 households were randomly selected, and members of the selected families were then interviewed by trained data collectors. Ultimately, 5,717 households with 16,908 individuals in urban areas and 6,900 households with 19,525 individuals in rural areas were enrolled and surveyed. The descriptive and socioeconomic characteristics of each income quintile are summarized in Table 1.

**Table 1:** Descriptive statistics of sampling data by income quintile

Region	Income quintiles	Per capita expenditures^a^	No. of surveyed households	No. of surveyed individuals
Urban	1 (poorest)	7,121.52	1,142	3,381
	2	12,324.44	1,144	3,371
	3	16,622.12	1,145	3,392
	4	21,989.18	1,143	3,382
	5 (richest)	37,709.70	1,143	3,382
	Subtotal	19,153.42	5,717	16,908
Rural	1 (poorest)	4,856.03	1,380	3,904
	2	8,836.33	1,380	3,911
	3	12,061.84	1,380	3,900
	4	16,302.20	1,380	3,914
	5 (richest)	30,785.07	1,380	3,896
	Subtotal	14,568.30	6,900	19,525

^a^All expenditures are presented in Chinese Yuan (CNY)

The survey collected extensive information about household socioeconomic characteristics and health care utilization, including the number of household members and their age, sex, education and employment status. Data on household expenditures included monthly housing costs and the amounts spent on clothing, food, water, transportation, education, electricity, fuel, communication, entertainment, travel, health care and other outgoings in the previous12 months. Per capita household expenditure adjusted by adult equivalence was used as the measure of living standard [16].

Information on per capita health care subsidy was obtained from two sources. The household survey included questions about outpatient visits, length of hospital stays, and the level of health care facilities that were visited. Outpatient visits were reported for a 2-week recall period prior to the survey and inpatient days were reported for a 12-month recall period. Administrative data from heath care facilities’ annual financial reports included information on government subsidies, outpatient visits, inpatient days and yearly outpatient and inpatient income at each level of health care facility. The household survey was confidential and the forms recorded no personal identifiers. The study was approved by the ethics committee of Nanjing Medical University. All participants provided written informed consent.

### Statistical analysis

We analyzed benefit incidence for both outpatient and inpatient care by level of health care facility. BIA was employed [17, 18], in which benefit incidence was presented as each quintile’s percentage share of total benefits and the concentration index (CI) was calculated [19, 20]. Thus, for example, if poor members of the population received a greater share of the government health subsidy than the rich, the CI was negative, indicating a pro-poor subsidy distribution [21].

Benefit incidence was calculated by multiplying the utilization rate of each type of service in each socioeconomic group by the unit subsidy of that service [22]. We calculated the unit subsidy by dividing the government health subsidy by the total number of outpatient visits or inpatient days at each level of health care facility. Data on government health subsidies for each level of health care facility were collected from local financial annual reports, but it was not possible to obtain separate data on subsidies for outpatient and inpatient care. Consequently, the relative proportions of outpatient and inpatient subsidies were calculated in our study. As noted above, patients in China receive government subsidies only when they consume medical care as outpatients or inpatients in health care facilities. Both outpatient and inpatient care were provided in the selected health care facilities, and we assumed that the proportion of subsidies received by outpatient and inpatient services could be estimated from the ratio of outpatient to inpatient income [14]. The unit subsidy at each health care facility level was calculated by dividing total service-specific subsidies by the total outpatient visits or inpatient days. The subsidy for each individual was calculated by multiplying total health care utilization by the unit subsidy at each facility level.

The computation of the CI required a comparison of covariance between variables and household fractional ranks according to ability to pay (ATP) [23]. The value of household expenditure was used as the measurement of ATP. Adjustment is made for the size and age structure of the household through application of an equivalence scale to ATP. The scale used was:

1 $$ AE={\left(A+0.5K\right)}^{0.75} $$

where *A* is the number of adults in the household and *K* the number of children (0–14 years) [24].

Then, estimates of the CI were obtained using ordinary least squares (OLS) regression of the variables of ATP and government health subsidy on the fractional rank in the ATP distribution [25]:

2 $$ 2{\sigma}_R^2\left(\frac{z_i}{\gamma}\right)=\alpha +\beta {R}_i+\varepsilon $$

where *z*_*i*_ is the government health subsidy to individual i, and *γ* is an estimate of its mean. *R*_*i*_ is the household fractional rank according to the ATP distribution and *σ*^*2*^ is its variance. The OLS estimate of *β* is an estimate of the CI [25].In addition, a dominance test was carried out to ascertain whether the concentration curve of government subsidies at one health care facility level dominated the subsidies at another facility level [26].

## Results

**Fig. 1:**
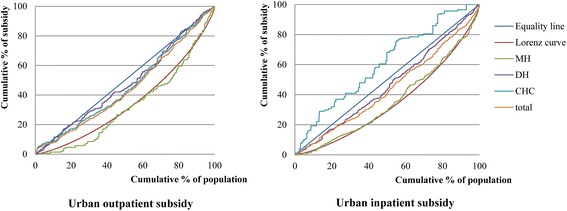
Concentration curves of government health subsidies in the urban population. Lorenz curves and cumulative concentration curves for government outpatient and inpatient subsidies in the urban population for 2012 data at different levels of health care facilities (CHC, DH and MH) are shown

In urban areas, government health subsidies were provided to CHCs, DHs and MHs, respectively. The distribution of government health subsidies in the urban population treated at the CHCs, DHs and MHs and stratified by income quintile is shown in Table 2. Concentration curves of the subsidies in the urban population at the different levels of health care facility are shown in Fig. 1.

**Table 2:** Distribution of government health subsidies by income quintile in the urban populations at different levels of health care facilities

Income quintiles	Per capita household expenditure	Community Health Centers		District Hospitals		Municipal Hospitals		Total	
		Outpatient care	Inpatient care	Outpatient care	Inpatient care	Outpatient care	Inpatient care	Outpatient care	Inpatient care
1 (poorest)	7.93 %	16.97 %	31.86 %	19.94 %	17.07 %	4.61 %	9.83 %	15.80 %	16.45 %
2	13.02 %	14.98 %	19.30 %	16.51 %	16.87 %	13.48 %	10.18 %	14.92 %	13.91 %
3	17.43 %	20.22 %	26.51 %	19.00 %	21.40 %	18.79 %	21.85 %	19.98 %	22.72 %
4	22.86 %	25.63 %	16.28 %	23.68 %	22.17 %	22.34 %	22.08 %	25.14 %	20.87 %
5 (richest)	38.77 %	22.20 %	6.05 %	20.87 %	22.49 %	40.78 %	36.06 %	24.17 %	26.05 %
Gini/concentration index	0.3066^**^ (0.3031 to 0.3101)	0.0795^*^ (0.0113 to 0.1478)	−0.2179^**^ (−0.3805 to −0.0554)	0.0465 (−0.0415 to 0.1345)	0.0752^*^ (0.0150 to 0.1355)	0.3456^**^ (0.2481 to 0.4430)	0.2883^**^ (0.2240 to 0.3527)	0.1068^**^ (0.0495 to 0.16414)	0.1237^**^ (0.0735 to 0.1739)
Weight		49.32 %	6.21 %	28.08 %	50.23 %	22.60 %	43.56 %	100 %	100 %

*Note*: 95 % confidence intervals of the concentration index presented in parentheses

**implies significant at 0.01

*implies significant at 0.05

**Fig. 2:**
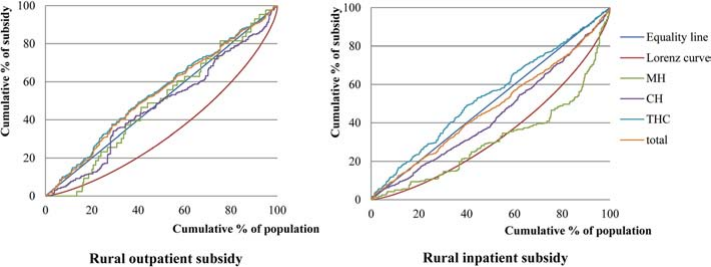
Concentration curves of government health subsidies in the rural population. Lorenz curves and cumulative concentration curves for government outpatient and inpatient subsidies in the rural population for 2012 data at different levels of health care facilities (THC, CH and MH) are shown

In rural areas, subsidies were provided to THCs and CHs, respectively. Because some rural patients seek medical care in MHs in urban areas, the distribution of government health subsidies for the rural population was analyzed within the scope of the THCs, CHs and MHs. The share of government health subsidies in the rural population treated at THCs, CHs, and MHs, stratified by income quintile, is shown in Table 3. Concentration curves of the subsidies in the rural population at the different levels of health care facility are shown in Fig. 2.

**Table 3:** Distribution of government health subsidies by income quintile in the rural populations at different levels of health care facilities

Income quintiles	Per capita household expenditure	Township health centers		County hospitals		Municipal hospital		Total	
		Outpatient care	Inpatient care	Outpatient care	Inpatient care	Outpatient care	Inpatient care	Outpatient care	Inpatient care
1 (poorest)	7.53 %	22.30 %	23.58 %	11.69 %	16.12 %	11.63 %	9.36 %	21.02 %	19.19 %
2	12.86 %	26.89 %	25.43 %	30.46 %	14.82 %	27.91 %	12.27 %	27.25 %	20.29 %
3	16.93 %	16.23 %	16.83 %	13.23 %	19.79 %	20.93 %	14.49 %	16.05 %	17.32 %
4	22.46 %	17.54 %	15.41 %	22.15 %	21.05 %	20.93 %	12.77 %	18.06 %	16.63 %
5 (richest)	40.22 %	17.05 %	18.74 %	22.46 %	28.22 %	18.60 %	51.11 %	17.61 %	26.56 %
Gini/concentration index	0.3250^**^ (0.3184 to 0.3317)	−0.0818^*^ (−0.1518 to −0.0119)	−0.0050^*^ (−0.0094 to −0.0005)	0.0567 (−0.0530 to 0.1665)	0.0084^**^ (0.0050 to 0.0118)	0.0271 (−0.1715 to 0.2256)	0.0252^**^ (0.0172 to 0.0331)	−0.0659^*^ (−0.1285 to −0.0034)	0.0036^*^ (0.0008 to 0.0065)
Weight		62.54 %	30.32 %	32.15 %	57.73 %	5.31 %	11.95 %	100 %	100 %

*Note*: 95 % confidence intervals of the concentration index presented in parentheses

**implies significant at 0.01

*implies significant at 0.05

In urban populations, of all types of health care provided at the different levels of facilities, we found that the only CI with a statistically negative value was that for inpatient care in CHCs (−0.2179), indicating that the poor received a greater proportion of government health subsidies than the rich when they sought medical care at CHCs. The CI values for all other types of health care were not negative, indicating that all medical services at all levels of health care facilities other than inpatient care at CHCs were not pro-poor. The inpatient CI values for DHs (0.0752) and MHs (0.2883) were statistically positive, implying that government health subsidies in those facilities were concentrated among rich patient populations. The outpatient CI values for CHCs (0.0795) and MHs (0.3456) were both statistically positive. The outpatient CI value for DHs was positive (0.0465), but it was not significantly different from zero. Accordingly, the hypothesis of proportionality cannot be rejected for DHs. The overall CI values for outpatient (0.1068) and inpatient (0.1237) care at all levels of health care facilities for the urban population were statistically positive.

In rural populations, the CI value for outpatient care in THCs was statistically negative (−0.0818), indicating that the poor who sought outpatient care in THCs received a greater proportion of subsidies than the rich. On the other hand, outpatient CI values were positive in both CHs (0.0567) and MHs (0.0271), but not significantly different from zero, which means that the hypothesis of proportionality cannot be rejected for outpatient care in CHs and MHs. The CI value for inpatient care in THCs was statistically negative but close to zero (−0.0050), suggesting that the subsidy for inpatient care was slightly pro-poor. CI values for inpatient care in both CHs (0.0084) and MHs (0.0252) were statistically positive, suggesting that government health subsidies were concentrated among the wealthy. Overall, the CI values for all levels of health care facilities in the rural population were statistically negative (−0.0659) for outpatient care and statistically positive for inpatient care (0.0036).

We tested the relative progressivity of concentration curves for subsidies at different health care facilities using dominance methods. For outpatient care in the urban population (see Table 4), the concentration curve for government health subsidies in CHCs dominated the curves for the subsidies in MHs. There were no significant differences between the curves for the subsidies in CHCs and DHs, but the curve for subsidies in DHs dominated the curve for MHs. For inpatient care in the urban population, the concentration curve for government health subsidies in CHCs dominated the curves for subsidies in both DHs and MHs, and the curve for the subsidies in DHs dominated the curve for MHs.

**Table 4:** Tests of dominance between the concentration curves for subsidies in the urban populations at different levels of health care facilities

	Outpatient care		Inpatient care	
	District hospitals	Community health centers	District hospitals	Community health centers
Municipal hospitals	D	D	D	D
District hospitals		Non-D		D

*Note*: “D” indicates that the concentration curve of the row subsidy dominates (is more progressive than) that of the column subsidy. “Non-D” indicates that non-dominance between the concentration curves cannot be rejected

For outpatient care in the rural population (see Table 5), the concentration curve subsidies in THCs dominated the curves for subsidies in both CHs and MHs. There was no significant difference between the subsidy curves in CHs and MHs. For rural inpatient care, the concentration curve for THCs subsidies dominated the curves for the subsidies in both CHs and MHs, and the curve for CHs subsidies dominated the curve for MHs. In general, dominance testing indicated that government health subsidies at the primary level of care—CHCs in urban areas and THCs in rural areas—were the most progressive, whilst the subsidies at the tertiary level of care (MHs) were the most regressive.

**Table 5:** Tests of dominance between the concentration curves for subsidies in the rural population at different levels of health care facilities

	Outpatient care		Inpatient care	
	County hospitals	Township health centers	County hospitals	Township health centers
Municipal hospitals	Non-D	D	D	D
County hospitals		D		D

*Note*: “D” indicates that concentration curve of the row subsidy dominates (is more progressive than) that of the column subsidy. “Non-D” indicates that non-dominance between the concentration curves cannot be rejected

## Discussion

So, to what extent is the distribution of government health care benefit subsidies in East China equitable? The present study found that the subsidies were pro-poor for inpatient care in primary health care facilities located in urban areas. However, subsidies of all other types of health care in urban areas at any facility level were not pro-poor. In rural areas, subsidies for both outpatient and inpatient care were pro-poor at primary health care facilities. Conversely, subsidies of inpatient care at the secondary- and the tertiary-level care facilities were pro-wealthy, and the hypothesis of proportionality could not be excluded for outpatient care although their CI values were positive. Dominance testing showed that primary health care facilities were more progressive than the secondary and tertiary care facilities in both urban and rural areas. Why then do primary health care facilities have better equity performance than the other facilities?

Since China does not have a gatekeeper requirement in the health sector, patients are free to choose any level of health care facility as their first-contact hospital. Moreover, Chinese patients traditionally prefer to seek medical care at higher levels of hospitals, even for illnesses that could easily be treated at a primary health care facility. On the other hand, for many years, primary health care facilities in China were not well run, largely because qualified medical personnel were not willing to work in lower-level hospitals but also because of lack of medical equipment. It therefore became common in China for patients to wait in long queues at specialty and general hospitals, even for the treatment of common ailments. However, these hospitals charged much higher prices for medical services, which meant that the OOP expenditures were very high, especially for poor patients. As mentioned earlier, patients were not entitled to benefit from government health subsidies if they did not undergo medical care in health care facilities. In other words, the subsidies were not available to lower-income groups who could not afford medical services. Thus, the distribution of government health subsidies among populations featuring different incomes was not equitable. Confronted with this situation, the Chinese government took steps to correct these problems by increasing the government healthcare budget, providing technical support and qualified medical staff to primary health care facilities and setting higher rates of health insurance reimbursement in lower-level hospitals.

The data in Table 6 show that per capita government health subsidies at primary-level health care facilities were much higher than those at secondary- and tertiary-level facilities. Higher government subsidies paid to primary health care facilities led to acceptable prices for medical care and wider access of medical care, especially for the poor [27]. One result of these changes was a decrease in OOP expenditures at primary-level health care facilities to levels much lower than at the secondary- and the tertiary- level facilities. The increased allocation of government health resources to primary health care facilities and the decrease in OOP expenditure not only widened access, but also influenced the treatment options that patients could choose. The result, in all probability, was to change the share of care-seeking behavior at the different levels of health care facilities among China’s different socioeconomic groups.

**Table 6:** Survey data on health care utilization and per capita subsidy by income quintile

Region	Income quintiles	Outpatient OOP^a^			Per capita outpatient subsidy			Patients in facility for outpatient care (%)			Inpatient OOP^a^			Per capita inpatient subsidy			Patients in facility for inpatient care (%)		
		CHCs (THCs)	CHs (DHs)	MHs	CHCs (THCs)	CHs DHs)	MHs	CHCs (THCs)	CHs (DHs)	MHs	CHCs (THCs)	CHs (DHs)	MHs	CHCs (THCs)	CHs (DHs)	MHs	CHCs (THCs)	CHs (DHs)	MHs
Urban	1 (poorest)	83.30	618.29	434.29	1725.46	235.95	575.73	16.90	20.73	4.04	2092.17	3505.45	6499.97	2343.38	353.42	1044.98	33.33	18.64	8.06
	2	90.49	355.49	340.16	1611.87	221.45	517.82	15.97	18.29	13.13	2228.57	3714.23	4681.33	2332.38	336.22	703.38	20.29	19.35	12.40
	3	143.67	403.74	563.92	1649.22	229.39	481.48	21.06	20.33	19.70	1582.11	4316.59	4772.91	2360.49	415.05	808.74	27.54	19.89	23.14
	4	70.99	593.06	409.94	1847.36	269.62	429.24	23.84	21.54	26.26	1223.60	4522.27	5776.60	2753.90	364.26	855.36	14.49	23.48	22.11
	5 (richest)	147.61	477.11	1219.01	1716.86	268.03	558.14	22.22	19.11	36.87	1933.33	7857.05	10780.42	3409.59	465.53	900.45	4.35	18.64	34.30
	Subtotal	108.52	494.20	733.89	1718.41	245.35	504.60	100.00	100.00	100.00	1846.61	4756.98	7183.04	2451.71	385.79	856.48	100.00	100.00	100.00
Rural	1 (poorest)	109.13	253.00	467.20	1329.39	238.16	354.30	22.17	13.76	13.89	1437.93	5336.49	18365.87	1502.85	383.95	957.44	21.46	15.80	9.68
	2	113.92	562.08	1140.91	1506.90	364.99	386.51	23.58	23.39	30.56	1493.34	3742.90	8832.35	1530.50	328.24	1107.88	22.73	17.00	10.97
	3	87.84	635.40	2729.14	1212.87	231.00	455.52	17.69	16.06	19.44	1647.95	5253.22	11450.00	1402.68	387.00	1010.70	16.41	19.39	14.19
	4	138.03	372.33	885.67	1244.51	276.28	531.45	18.63	22.48	16.67	1400.81	5130.09	8370.00	1112.89	390.17	783.59	18.94	20.05	16.13
	5 (richest)	154.61	754.28	2057.14	1257.36	258.98	404.91	17.92	24.31	19.44	1837.12	7537.34	19253.95	1253.15	382.80	1018.47	20.45	27.76	49.03
	subtotal	119.99	535.39	1491.78	1321.92	280.31	423.19	100.00	100.00	100.00	1559.62	5613.86	15079.41	1367.76	376.02	983.61	100.00	100.00	100.00

*Note*: *CHs* county hospitals, *CHCs* community health centers, *DHs* district hospitals, *MHs* municipal hospitals, *OOP* out-of-pocket expenses, *THCs* township health centers

*Source*: Author’s calculations from the present study’s household survey

^a^All expenditures are presented in CNY (real prices and costs as at 2012)

As shown in Table 6, the poor chose grassroots (i.e., primary-level) health care facilities more often than the rich did. Using inpatient care in urban areas as an example, 33.33 % of the poorest and 20.29 % of the poorer sought care in CHCs, compared with 8.06 % and 12.40 % in MHs. In addition, the reimbursement rate of public health insurance was higher in primary-level health care facilities than in the secondary and the tertiary level facilities in China. This policy encouraged patients, especially poor ones, to treat common ailments in lower-level hospitals. Thus, the frequent use of medical services by people in poor socioeconomic quintiles and the higher allocation of government resources to primary health care facilities changed the utilization of health care facilities, resulting in an enhanced opportunity for poor and vulnerable groups in obtaining subsidies. Consequently, the distribution of government benefits at primary health care facilities became more equitable than the distribution at secondary- and tertiary-level facilities.

We also found that equal subsidization—that is, rich and poor patients receiving the same amount of subsidy for the same medical care—was a potential threat to equity in government health care benefit distribution. The wealthy are more able to afford health care services and therefore have more opportunity to obtain government health subsidies. This explains why the benefit distribution of urban outpatient services was inequitable at primary-level facilities. After the government introduced policies that encouraged patients to seek medical care in lower-level hospitals, urban residents began to feel more confident in seeking treatment at primary care facilities and recognized that they did not need to go to higher-level hospitals. We thus found that more outpatient CHC visits were made by rich patients than by poor patients. That is to say, compared with the poor, the wealthy had many more opportunities to receive government health subsidies, resulting in an inequitable benefit distribution for urban outpatient services.

Some limitations of our study must be acknowledged. Undertaking BIA depends on the availability of data. Government health subsidies by type of health care were not available in China’s governmental financial reports. We therefore made the assumption that the proportion of subsidies received by outpatient and inpatient services could be estimated from the ratio of outpatient to inpatient income. We also assumed that all outpatient and inpatient services (maternity, cardiac and orthopedics, in the case of inpatient care; similarly for outpatient care) were equally subsidized, respectively. It would be of value to develop more refined measures to calculate the proportion of subsidies by type of health service use, in order to more closely assess unit subsidy with respect to different types of health care.

## Conclusions

In China, the distribution of government health subsidy benefits to primary-level health care facilities shows better equity performance than with secondary- and tertiary-level facilities. Key factors for improving equity have included the increased government budget allocations, the provision of technical support and qualified medical staff to lower-level hospitals and higher health insurance imbursement rates. These changes have resulted in decreased OOP expenditure and a more frequent use of health services at primary health care facilities by patients in poor socioeconomic quintiles. However, an increase in the proportion of the rich seeking medical care in grassroots hospitals has resulted in a decrease in the equity of benefit distribution. Therefore, a differential allocation of healthcare subsidies among the different socioeconomic groups in China is needed to make the distribution of government health care benefits more equitable still.

## Abbreviations

ATP: ability to pay
BIA: benefit incidence analysis
CHs: county hospitals
CHCs: community health centers
CI: concentration index
DHs: district hospitals
MHs: municipal hospitals
OLS: ordinary least squares
OOP: out-of-pocket
THCs: township health centers
VC: village clinics
